# Qualitative assessment of programmatic constraints in delivery of effective interventions for improving maternal nutrition in Bangladesh

**DOI:** 10.1136/bmjnph-2021-000395

**Published:** 2023-02-15

**Authors:** Md Golam Rasul, Mahamudul Hasan, Daluwar Hossain, Fariha Haseen, Subhasish Das, Tahmeed Ahmed

**Affiliations:** 1 Nutrition and Clinical Services Division, International Centre for Diarrhoeal Disease Research Bangladesh, Dhaka, Bangladesh; 2 Department of Public Health and Informatics, Bangabandhu Sheikh Mujib Medical University, Dhaka, Bangladesh

**Keywords:** nutrient deficiencies, nutrition assessment

## Abstract

**Introduction:**

Maternal undernutrition is highly prevalent in most of the developing countries. Prevalence of both extremes of maternal malnutrition (undernutrition and overweight/obesity) are common in those countries. For Bangladesh, the scenario is not different. The Government of Bangladesh recognises maternal nutrition as a public health priority and addresses the issue in its policies and programmes. We identified and analysed the existing maternal nutrition programmes and determined the bottlenecks in implementing the programmes in Bangladesh using qualitative approach.

**Methods:**

We followed a qualitative research approach and conducted 25 key informant interviews with the programme managers and policymakers, 10 in-depth interviews with the service providers and six focus group discussions with the pregnant women to identify the constraints of programme implementation. We analysed data using thematic and inductive approaches of qualitative research methods.

**Results:**

We have found that successful implementation of maternal nutrition intervention was being hampered by both the demand and supply side issues. On the demand side, major constraints were financial inability of the families to avail maternal nutrition-related services, ignorance of the family members and cultural barriers of using maternal nutrition-related services. Lack of priority and heavy workload of the service providers, lack of human resources, poor monitoring system, lack of medicine to supply and incoordination have been identified as major supply-side constraints in providing maternal nutrition-related interventions in Bangladesh.

**Conclusion:**

Both supply side and demand side issues are responsible for the existing bottlenecks in implementing maternal nutrition-related programmes in Bangladesh. Findings of this study will help the policymakers to learn about the programmatic constraints regarding maternal nutrition services in Bangladesh.

**WHAT IS ALREADY KNOWN ON THIS TOPIC**

- In Bangladesh, maternal undernutrition is still a burden, and the Government of Bangladesh recognises maternal nutrition as a public health priority and addresses the issue in its policies and programmes.
- Programmatic constraints of maternal nutrition interventions in Bangladesh have never been identified.

**WHAT THIS STUDY ADDS**

- We have found that incoordination among various government organisations, poor planning in health service delivery, heavy workload of the health service providers and poor service delivery structure hampers maternal nutrition service delivery in Bangladesh.
- Still, misconceptions regarding pregnancy and childbirth persist in different areas of Bangladesh. Lack of family support restrains the mother from receiving maternal nutrition-related services in many areas.

**HOW THIS STUDY MIGHT AFFECT RESEARCH, PRACTICE OR POLICY**

- Study findings will provide information to the policymakers about the programmatic constraints regarding maternal nutrition services in Bangladesh.
- Findings from this study will help the policymakers to identify the areas where more coordination is required.
- This study will help the policymakers understand the need for a new intervention to bring more urban women under the coverage of service delivery umbrella.

## Background

Maternal nutrition refers to the nutritional needs of women during antenatal and postnatal periods and it may extend to the periods before (ie, during adolescence)^1^ and after conception. Adequate nutrition is a prerequisite for good maternal health, quality of life and national productivity.^2^ Undernutrition and overnutrition (overweight and obesity) can impose significant risks on maternal health and associated pregnancy outcomes. A woman of poor nutritional status (indicated by a low body mass index, short stature, anaemia or other micronutrient deficiencies) possesses heightened risk of obstructed labour, delivering a baby with low birth weight, producing breast milk with poor nutritional composition and dying from postpartum haemorrhage.^3^ Maternal overweight and obesity, however, can lead to several maternal (gestational diabetes, pre-eclampsia) and fetal/childhood complications (stillbirth, congenital anomalies and autism).^4^ To that end, without addressing the constraints of proper maternal nutrition, the vicious cycle of trans-generational transmission of malnutrition, chronic diseases and poverty will be perpetuated.

Maternal undernutrition is a persistent phenomenon in most developing countries. About 20% of women in sub-Saharan Africa, South-Central and South-Eastern Asia have a body mass index of less than 18.5 kg/m².^3^ Chronic energy and micronutrient deficiencies are prevalent, especially in South-Central Asia, where more than 10% of women aged 15–49 years are shorter than 145 cm.^3^ The prevalence of overweight and obesity has also increased substantially in developing countries. In China, 5.0% of women were obese in 2013, compared with 4.2% of women in India.^4^ Adolescents are equally vulnerable to malnutrition and with the high rate of adolescent pregnancy in this region, the risk of complications during pregnancy and poor birth outcomes is also high. Similar scenario can be found in South Asia. In India, 32% of adolescents were stunted and in Nepal the rate was 47%.^4^ Low body mass index is also highly prevalent in these two countries (53% in India and 36% in Nepal). In India, a recent report shows that 55.8% of adolescents aged 15–19 years and 56.7% of women aged 20–24 years were anaemic.^4^ Overweight and obesity in adolescents have also increased in developing countries (8.4% in 1980 to 13.4% in 2013).^5^ Because of the established health risks and substantial increases in prevalence, maternal malnutrition has become a major global health challenge.

In Bangladesh, the burden of maternal malnutrition is no different from neighbouring countries. The Government of Bangladesh (GoB) recognises maternal nutrition as a public health priority and addresses nutrition in its policies and programmes. GoB is committed to achieve the targets of Sustainable Development Goal by ending all forms of malnutrition by 2030.^6^ This goal depends on achieving, by 2025,^7^ the World Health Assembly global targets on stunting and wasting in children under 5 years of age and addressing the nutritional needs of adolescent girls, pregnant and lactating women and older persons. In line with the global agenda, the Ministry of Health and Family Welfare, Government of Bangladesh, has implemented the Health Population and Nutrition Sector Development Program.^8^ In contrast, the issue of assessing coverage and qualities of interventions as their impact on maternal health and nutrition in the country is still indecisive. Potential barriers to the usage of these services require further exploration. A study in India revealed that systematic weakness, logistic gaps, resource scarcity and poor usage hamper maternal nutrition intervention.^9^ A similar study in Nigeria showed that weak advocacy for nutrition, lack of coordination among government and non-governmental actors contribute to low prioritisation.^10^

We conducted a study to identify the maternal nutrition interventions and the barriers for implementing such interventions in Bangladesh. We believe that barriers explored in this study would provide a basis for improving nutrition interventions that would affect long-term quality of life and reduce mortality, morbidity and healthcare costs for both mother and child. Other countries would also benefit from such a study as the findings will provide an excellent platform for improving maternal nutrition in low-resource settings. Hence, the primary objective of this study was to analyse the existing maternal nutrition programmes to identify programmatic constraints in delivery of effective interventions for improving maternal nutrition in Bangladesh. Our secondary objective was to identify service delivery barriers for implementing maternal nutrition interventions and determine the barriers to receiving maternal nutrition interventions in the community.

## Methods

### Study design

We conducted a cross-sectional study from January to December of 2018 to identify maternal nutrition-related programmes and barriers to service delivery and receiving of services using qualitative methods. We did an extensive desk review and listed maternal nutrition services provided by the government and non-government service providers. The lists can be found in online supplemental tables 1 and 2. Then we identified the potential stakeholders responsible for implementing these nutrition programmes in Bangladesh. Following that, 25 key informant interviews (KII) were conducted to identify the barriers to implementation, 10 in-depth interviews (IDI) were conducted with the service providers to identify the service delivery barriers and 6 focus group discussions (FGDs) were conducted to identify the extent of programme receiving barrier among the pregnant women. Informed written consent was taken from the study participants before conducting the interviews.

10.1136/bmjnph-2021-000395.supp1 Supplementary data

### Study setting

Data was collected from both urban and rural settings of three divisional cities—Dhaka, Khulna and Sylhet. Sylhet was selected because of its highest rate of maternal undernutrition and Khulna was selected because of its lower prevalence of undernutrition but higher prevalence of overweight and obesity than other divisions.^11^ The capital of the country, Dhaka, was selected as most of the nutrition service-providing organisations and governing agencies are situated in this city. These three cities provided diverse contexts regarding health and nutrition infrastructures. Selection of urban and rural communities was based on our desk review and recommendation from initial key informants working at policy or management level.

### Data collection tools and participant selection

We followed a purposive sampling method for selecting the study participants. For KII, we selected the persons having understanding and experience to find out challenges and bottlenecks in delivering nutrition services for women or mothers. For IDI, we selected the service providers who had hands-on experiences in maternal nutrition-related service delivery. Barriers to service delivery were identified through the interviews. For determining the barriers to receiving maternal nutritional interventions, FGDs were conducted with mothers visiting for antenatal checkups in urban and rural primary healthcare settings of selected study areas. All interviews were recorded after taking permission from the participants, and a team of two interviewers conducted the interview session where one of them facilitated the interview/ FGD and the other one took notes.

### Data analysis

We analysed data using thematic and inductive approaches of qualitative research methods. All the interviews were audio-recorded and transcribed in Bengali. Then, transcripts were manually reviewed and coded for related constraints of maternal nutrition-related services in Bangladesh. Transcripts were double coded, and disagreements were solved through discussion. Analysis was guided by the evaluation objective. Study team read the raw data multiple times and interpreted the data to carry out the analysis. The primary outcome of analysis was to develop categories from the raw data to models or frameworks. In this model, the evaluator identifies key themes during the coding process. During analysis, prominent themes and subthemes were identified and the common patterns that emerged through all interviews were marked. To explain the meanings and to create new insights for further investigation, patterns were identified from the transcription based on the themes. Finally, we prepared a matrix based on the coding. The quotes were translated into English verbatim during developing the manuscript.

### Patient and public involvement

Patients or the public were not involved in the design, or conduct, or reporting, or dissemination plans of our research.

## Result

### Study participants

Table 1 presents the characteristics of FGD, KII and IDI participants. A total of six FGDs, 25 KIIs and 10 IDIs were conducted. The participants of FGDs had variable age groups, pregnancy number, trimesters and educational qualifications. Also, participants were from both urban and rural areas. All the IDI participants were female service providers and 76% of the KII participants were male service providers. Among the KII participants 14 were from government implementers and 11 were from different non-governmental organisations who were implementing different maternal nutrition-related programmes in Bangladesh.

**Table 1:** Characteristics of FGD, KII and IDI participants

Distribution of FGD participants (N=50)	n	%
Mothers’ age		
20 and less	18	36
21–25 years	15	30
26 and above	17	34
Number of pregnancies		
1	18	36
2	18	36
3	10	20
More than 3	4	8
Duration of the pregnancy		
First trimester	9	18
Second trimester	16	32
Third trimester	25	50
Residence		
Rural	25	50
Urban	25	50
Education of the mothers		
Primary completed	14	28
Secondary completed	26	52
Higher	10	20

Distribution of KII and IDI Participants	KII n (%), n=25	IDI n (%), n=10
Location		
Dhaka	19 (76%)	3 (30%)
Sylhet	3 (12%)	3 (30%)
Khulna	3 (12%)	4 (40%)
Gender		
Male	19 (76%)	–
Female	6 (24%)	10 (100%)
Working affiliation		
Government policymakers	5 (20%)	
Government implementer	5 (20%)	7 (70%)
Non-government implementer	4 (16%)	3 (30%)
Non-governmental organisations	11 (44%)	

FGD, focus group discussion; IDI, in-depth interview; KII, key informant interview.

### Barrier of maternal nutrition intervention in Bangladesh

#### Barriers identified from demand side

We identified the factors affecting the receipt of services from FGDs with service receivers. These factors that we have identified are: financial factors, household factors and barriers to service delivery from the service centre. The framework is presented in table 2.

**Table 2:** Themes of barriers identified from the demand side

Barrier	Outcomes	Impact
**Financial factors** Irregular consultation and medicine purchase. Purchase of child centric foods. Less food consumption.		
**Household factors** Dependency on family members for service receiving. Elderly member does not allow to take medicine. Obliged to consume less food. Heavy workload. Lack of interest for service receiving from the family members.	Poor maternal nutrition service usage.	Poor nutritional status of pregnant women and poor pregnancy outcomes.
**Health/nutrition service factors** Insufficient medicine supply. Distance of service centre. Insufficient maternal nutrition services provision. Provide limited service from the service provider’s end.		

### Financial factors

Most of the pregnant women stated that they could not use services from the healthcare centres because of their poor economic conditions. Sometimes they missed one or two check-ups because their family did not have enough money to go for checkups. The situation was more common in the urban area as none of the pregnancy-related services was free of cost at any urban healthcare centres. One pregnant woman mentioned:

No, they didn’t give me any medicine yet. They [service providers] called me to come to the service center. But I could not come due to lack of money. I didn’t have enough money in my hand. My husband will get his salary after 10th of the coming month. Then I have to take the medicine.

Services from government healthcare centres were free of cost in rural areas. But medicine was not entirely free anywhere. Rural mothers received a limited amount of medicine items from the community clinics, but urban women hardly got any free treatment from the nearby service centres. In the rural areas, most of the mothers consumed only free medicines provided by the healthcare providers. And, when they consumed the doses of free medicines, they could not buy new medicines and had to wait until the next visit to get free medicines again. Most pregnant women stated that their families could not buy nutritious foods for them during their pregnancy. In most of the families, food purchase was usually child-centric, and mothers were more focused to feeding their children.

### Household factors

Within the household, treatment-related decisions were usually taken by the husband or other elderly members of the family and women were highly dependent on their husbands or mothers-in-law to decide for pregnancy check-ups or for taking nutritious foods during pregnancy. Sometimes elderly members of the family did not allow the pregnant mother to eat healthy food or any other medicine. The two most common conceptions identified were ‘medicine during pregnancy is harmful to the child’ and ‘eating nutritious food would increase the weight of the newborn child, and it might create complexities during birth of the child’. FGD participants from the Sylhet area stated:

My mother-in-law told me not to eat much; it would make my child bigger and create problems during delivery. The health worker always asks me to eat more nutritious food. But I can’t defy my mother-in-law’s advice; I am new in this family; if any problem arises, it will affect my paternal family and me as well.

Another pregnant woman stated:

‘During my first pregnancy, my mother-in-law did not allow me to take any medicine, even if I felt sick. She told me that it would harm the baby. Due to birth injury, my baby is autistic, and now for my 2nd pregnancy, I am having a regular check-up and taking medicines properly as advised by my service provider. I feel better now.

The respondents also reported that family responsibilities and cultural barriers hampered better nutritional practices during pregnancy. One common phenomenon in rural areas was that women usually took lunch or dinner as the last person of the family. As a result, sometimes they had to eat less or sometimes there remained no food at all for them to eat. In most of the cases, they did not get any special treatment from family members as a pregnant woman.

I cook in the morning, after cooking, I do all of my household works and help my husband to get prepared for his office by 8 am. Then I complete all my household works, take my bath, and then I take my meal. Sometimes I couldn’t take my breakfast. There are five members in my family. Every time, I take my meal after everyone has eaten.

Similarly, the service providers indicated that they had to face different types of non-cooperation within the family to convince the mothers to receive maternal nutrition-related services and to maintain good nutritional practices. Especially the mothers-in-law and other elderly persons from the family discouraged the mothers from taking nutrition-related services and practicing those.

### Service provider’s factors

During FGDs mothers stated that more attention was given to child’s health and nutrition than pregnant women in service centres. Service providers did not prioritise maternal nutrition activities and eventually nutrition-related services and nutritional practices were hampered.

Some of the interviewed mothers could not come to the healthcare facility as some healthcare centres were far from their home and transportation was not easily available. Also, few of the mothers did not want to go to the service centres because most service centres did not provide adequate services for pregnant women. Community clinic is the primary service centre in the rural area. Most of the rural pregnant women visited community clinics for healthcare services. In our study, no community clinics were found to provide maternal nutrition-related counselling or services. Mothers got some supportive treatments from there, but the quantity of the medicine was not sufficient for them. Besides this, rural women did not want to go to the upazila health complex because this facility was overcrowded with patients. As a result, doctors could not examine them and provide health messages appropriately. Though urban mothers received some nutrition-related messages from the healthcare providers, the situation was worse in rural areas as none of the rural mothers received any nutrition-related services or messages from any healthcare providers. One mothers from the rural site stated:

I do not get any service from here. If we come here, the service providers give me some medicine. If I tell her about my problem, the service provider advises me to visit the upazila health center.’

On the contrary, service providers also mentioned some barriers regarding the service receivers that echoed our findings. One common trend that was mentioned by the service providers is, in Bangladesh, many pregnant mothers move from town to village or from in-laws’ home to parent’s house during pregnancy. When a mother moves from one catchment area to another, she misses the service she is supposed to receive as sometimes it becomes tough for the healthcare providers to track and serve the pregnant mothers.

### Barriers identified from supply side

We identified service provider and service centre-related factors from in-depth interviews of the healthcare providers and key informant interviews of programme managers. The framework is presented in table 3.

**Table 3:** Themes of barriers identified from the supply side

Barriers	Outcome	Impact
**Service provider’s factors** Inexperienced service providers. Heavy workload. Lack of staffs and large coverage area. Lack of importance for maternal nutrition-related services.		
**Service centre factors** Lack of medicine supply. Provider’s behaviour. Service centre environment. Service costs. Unplanned community clinic. Lack of coordination. Lack of monitoring.	Hampers service quality and availability.	Poor maternal nutrition service delivery.
**Demand side factors** Lack of family support. Lack of awareness/prioritisations. Movement of mother.		

### Service provider’s factors

Primary level service providers of both health and family planning departments had to focus on activities other than maternal nutrition-related services such as immunisation programmes, child health, family planning activities, family planning method distribution. Such activities did provide some indirect messages about child nutrition, but they usually did not offer any maternal nutrition-related services or messages. Also, service providers did not carry any instruments to assess the nutritional status of the mothers during field visits. As a result, no need-based nutrition-specific instructions were delivered. One of the service providers stated:

We meet the mother regularly, but we do not have any instrument to assess their nutritional condition. We provide them some general messages.”

Another finding behind poor service delivery was lack of training of service providers on maternal nutrition-related services. During the in-depth interview sessions service providers stated that they did not receive any hands-on training to assess the nutritional status of mothers; a few of them received general training on nutrition provided by the Institute of Public Health and Nutrition, Bangladesh. One of the key informants mentioned the importance of training:

What is the reason behind Expanded Programme on Immunization (EPI) becoming so successful in Bangladesh? EPI has been a great wonder for the last 20 years. There are so many training programs on EPI. Each of the field level staff gets training and refreshers on EPI.

Besides this, shortage of service providers is another barrier to ensure maternal nutrition-related services. According to the policy, one healthcare provider should be deployed for every 5000 people in rural areas.^12^ But practically that ratio was two-to-three times higher in every catchment area. Some service providers have to provide services to additional areas. Such practice increased their workload and decreased the quality of service they rendered. One health worker mentioned:

There should be one Health Assistant for every 5000 people. But in my area, there are around 15000 people. If I do my regular work, it requires three times more to complete each cycle of visits. Moreover, I oversee an additional area. It requires around 6 months to complete each cycle of visits, which I was supposed to do within 2 months. Now during my visit, if I get a mother at the fourth month of her pregnancy, in the second visit, in most cases, the mother already has delivered the baby. How can I ensure maternal nutrition services for the mother then?"

Prioritising the record-keeping and documentation processes is another constraint of providing good maternal nutrition-related services. One service provider mentioned:

Most of the time we face problem during counselling. We need to keep records and fill-up the reporting form which takes more time. So due to record-keeping or reporting, we can’t counsel the mother properly……I have many other tasks which I am not supposed to do."

### Service center factors

Lack of medicine was one of the leading constraints of ensuring maternal nutrition-related services. From FGDs and IDIs we found that most of the service centres could not provide adequate iron-folic acid (IFA) tablets to the mothers until they reached 6 months of their pregnancy. But pregnant women should get IFA tablets from the third month of their conception. According to a policymaker, one pregnant mother is supposed to get 60–90 IFA tablets during each visit to the healthcare centre. Instead, they got only 10–20 IFA tablets during every such visit. One policymaker stated:

We decided that service providers should deliver at least 60–90 IFA tablets to pregnant mothers at a time. Instead, if providers dispense IFA tablets only for 3/4 days, then obviously mother will not visit again after 3/4 days.

But in the community clinic, service providers did not get the opportunity to provide more than 10–20 tablets at a time. Moreover, locally influential people often take more medicines than required. One service provider said:

We give medicines for 2/3 days for general patients and 10/20 IFA tablets for pregnant mothers, but sometimes locally influential persons take more medicine using their social or political influences.

On the other hand, service providers face problems within the community if they provide different doses of medicine to different persons. That’s why they do not want to provide the instructed amount (60–90) of medicine to pregnant women. One service provider mentioned:

At the community clinics, Community Health Care Providers might suffer if she gives different doses to different patients. If CHCPs do that, they might lose their acceptances in the community."

Also, medicine supply in the community clinic was not regular. Sometimes, community clinics did not receive any supplies of medicines for pregnant women for 2–3 months. One of the community health workers mentioned:

Sometimes there is no supply of medicine for 4–5 months. We have to wait for medicine, and we do not know when it will be supplied again."

Primary healthcare providers and the service receivers—both shared their concerns on the environment of the service centres. They mentioned that most of the service centres (community clinics, upazila health complex) remained overcrowded during service hours. Hence, doctors could not serve the mother properly like they do in the private clinic. During FGDs pregnant mothers mentioned that taking services from government health facilities was for the poor people only, and those who had money would receive good services from private clinics. For this reason, members of middle-class households did not take services from government established healthcare centres while in some cases they could not afford the private clinic too.

Another major constraint for ensuring proper healthcare services was the lack of coordination among different government departments regarding procurement of medicine and supply chain management. Lack of coordination was visible as lengthy procurement processes created a medicine crisis at the service delivery point. One key informant mentioned the reason behind the protracted process of medicine procurement:

We have to complete the whole procurement system through Central Medical Stores Depot (CMSD). If we can provide logical and required papers to CMSD they will process it quickly. But we do not send papers for any single item; rather we order as a package. So, if any item from the package doesn’t get approved, the whole procurement process gets delayed.

Also, lack of coordination was found during medicine distribution among various healthcare centres. Every community clinic received the same amount of medicine irrespective of the size and population catchment area. Primary healthcare providers did not have any scope of providing requisition of their required number of medicines except dispensing the number of medicines they received from the centre.

‘They [centre] do not care about our demand. So, whatever amount I get, I manage the demand with that.

Catchment area distribution was not the same for every community clinic. Moreover, the current monitoring system for maternal nutrition-related services was not adequate to ensure proper services. One key informant from a non-government organisation mentioned:

Current register has been updated in 2015 for the last time. By this time, many antenatal care indicators have been changed. Some maternal health and nutrition-related component have been added. We tried to give our inputs whenever they [the government] intended to change the format.

## Discussion

The study was designed to identify potential constraints of implementing maternal nutrition-related services in Bangladesh. We identified constraints from both supply and demand sides. Financial problems, family pressure to consume less amount of food, lack of awareness about good nutritional practices, heavy workload and lack of family interest have been identified as significant constraints of receiving maternal nutrition-related services, affecting the successful implementation of maternal nutrition-related interventions in Bangladesh.

Many studies have reported that in low-income and middle-income countries, food consumption during pregnancy mostly depends on the family income status.^10 13 14^ Even in high-income countries, low- and middle-income families could not ensure nutritious foods for pregnant women.^15 16^ A study in Ethiopia stated that the poor economic status of the family was the leading barrier to provide healthy food consumption.^17^ Another study in Ethiopia showed that around 40% of the pregnant women had low dietary diversity.^18^ In our research, we also observed the similar result. Pregnant women and their family members were not aware of dietary diversity and the need to consume more food during pregnancy period. Usually, most household level food purchases were child-focused, and many pregnant women shared their foods with the child. Dietary habits of the mother during pregnancy is thought to be one of the important factors influencing the health of both the mother and her growing fetus.^19^ However, some mothers-in-law did not allow them to eat more during their pregnancy. One common misconceptions in rural areas of Bangladesh is that, eating more food during pregnancy would increase the size of the child which may further complicate the delivery. The tradition is also prevalent in various parts of the world. Earlier studies suggested that in India, Nepal, Pakistan, Indonesia, Laos and Senegal, mothers intentionally ate less during their pregnancy to avoid delivering a large child.^14 20 21^ Another study suggests that support from husband and other family members were particularly essential to ensure proper nutritional practices for the pregnant women.^22 23^

Pregnant women were unable to take their meals timely due to their heavy workload and cultural barrier. In Bangladesh, women of the households are the last person to dine.^24^ Our study findings also echo the same. Some of the FGD respondents mentioned that they could not eat on time due to heavy workload to complete the household chores. Some other respondents mentioned they had to wait until everyone completed their meal. A study in Tanzania noted that women had to compromise their meals due to heavy workload and could not eat an adequate amount of food as only a minimal amount of food was left after the family members completed taking their meals.^25^ Our study also documented that the pregnant women we surveyed rarely got any opportunity to take rest during the day, and they had to do all their household work which caused meal skip for one to two times a day. Previous studies indicate that adequate rest during pregnancy improves fetal weight and reduces the risk of pre-eclampsia or gestational hypertension.^26 27^

Lack of priority and heavy workload of the service providers, lack of human resources, poor monitoring system, lack of medicine supply and incoordination have been identified as major supply-side constraints of providing maternal nutrition-related interventions in Bangladesh. Bangladesh has implemented National Nutrition Policy 2015, Second National Plan of Action for Nutrition (2016–2025), National Food Policy Plan of Action (2008–2015), Seventh 5-Year Plan Bangladesh (FY2016 – FY2020) and Nutrition Background Paper 2015, Program Implementation Plan of HPNSP (2017–2022), National Nutrition Service Operational Plan (2017–2022) for promoting maternal nutrition targeting the improvement of the health and nutrition of the population.^28^ These policies emphasise nutrition and health education for all and established health and nutrition units in every upazila^29^ to motivate the mother to take nutritious foods and to ensure nutrition for all through behaviour change communication by providing counselling services at the family level,^30^ provision of iron, folic acid or multiple micronutrients supplements, promotion of the use of calcium during pregnancy as a supplement, ensuring nutrition counselling during antenatal care (ANC) and postnatal care (PNC) and micronutrient and food supplementation for all mothers.^31^ But our study found gaps between the policy and its implementation. According to a recent report only half (46%) of the women who gave birth in the last 3 years took iron tablets or syrup for at least 90 days during their pregnancy.^32^ Another study from Bangladesh reported per capita calcium consumption for women as 184 mg/day, which is far below the recommended intake of 1000 mg/day for working-age adults.^33^ In Bangladesh, the Directorate General of Health Services and the Directorate General of Family planning are the main responsible bodies for providing health and nutrition-related services at the root level. Health Assistants and Family Planning Assistants are designated for implementing health and nutrition care services and Community Healthcare providers at field level service delivery centre and Family Welfare Visitors are designated for delivering centre-based health and nutrition-related services. At the same time, they are responsible for implementing all the other activities of the health and family planning department (such as EPI, family planning method distribution), which is the cause of their heavy workload. As a result, activities related to nutrition-related service delivery became less prioritised and that eventually affected the field implementation of the policies taken by the GoB. According to the Bangladesh Health Situation Report 2019, around 25% of the domiciliary staff position was vacant.^34^ This shortage of service providers hampers the quality of service provided to the demand side. A study conducted in Iran indicated that proper nutrition message during antenatal check-up was an essential predictor of improving pregnant women’s nutritional status.^35^ Another study from Nepal reported that women were not receiving proper maternal nutrition-related services due to the busy schedule of the service providers, lack of resources and lack of adequate training of the healthcare providers.^36^ Beside the workloads, coordination among the respective bodies is another major constraint of maternal nutrition service delivery in Bangladesh. In our study we found that three government institutions are implementing maternal nutrition-related services in Bangladesh. Lack of internal coordination among them, lack of proper data collection and reporting and lack of prioritisation from the service providers were affecting the nutrition service delivery. According to a previous report 22 ministries were implementing nutrition sensitive interventions in Bangladesh and they suffered from lack of coordination regarding monitoring, reporting and data management among them.^37^ Our study findings are also aligned to this.

**Figure 1:**
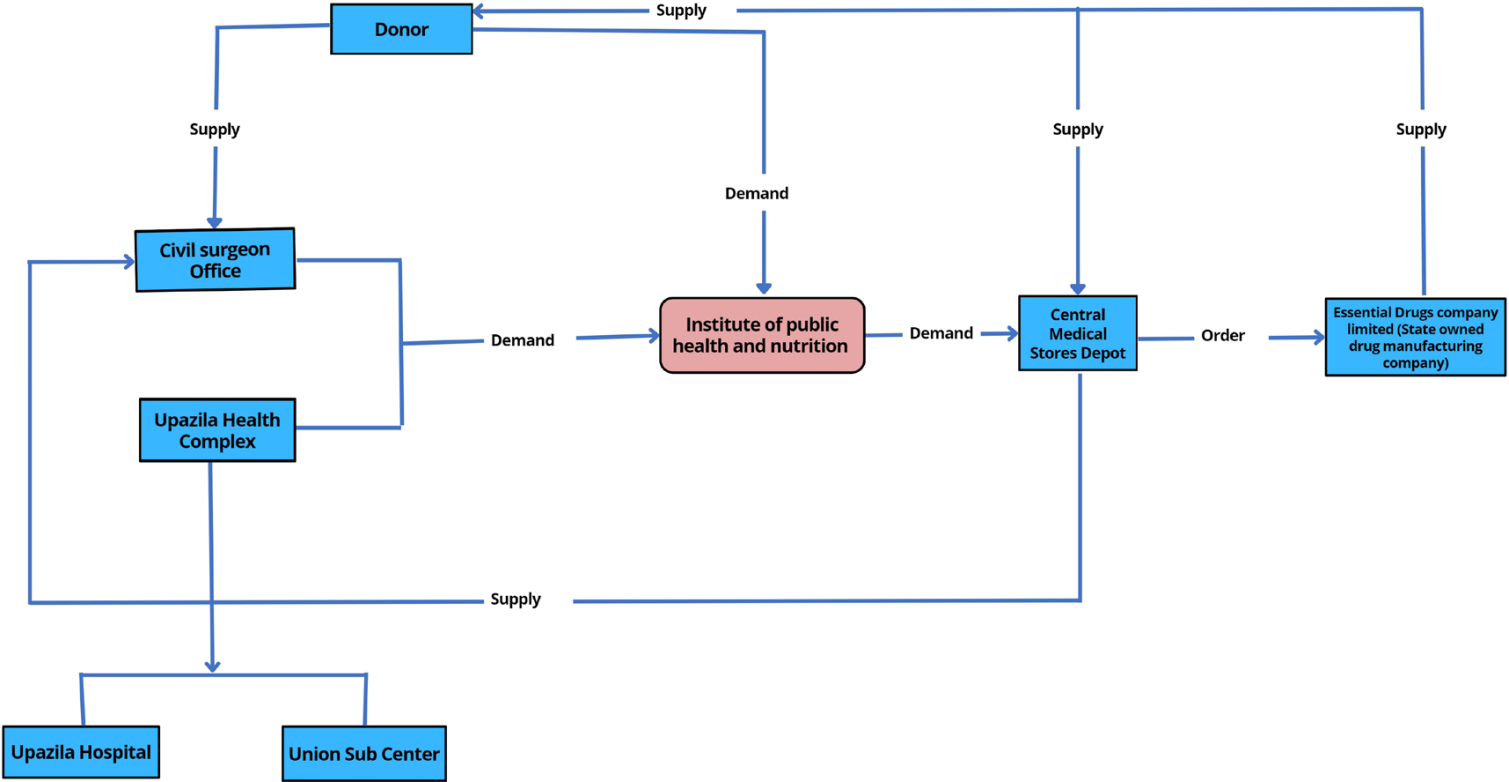
Iron-folic acid and calcium supply chain management of Institute of Public Health and Nutrition through Central Medical Store Deport.

**Figure 2:**
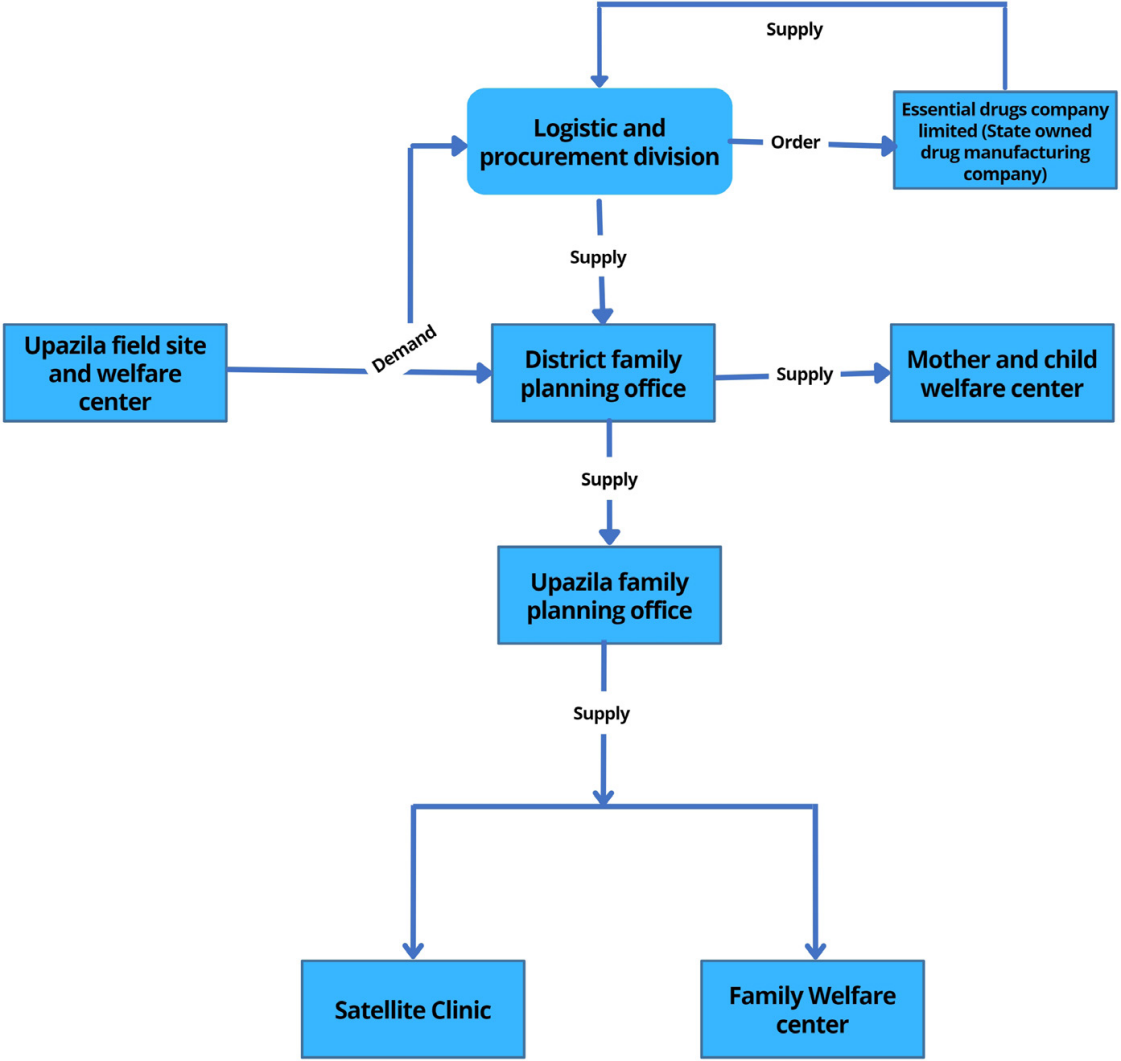
Iron-folic acid and calcium supply chain management of Directorate General of Family Planning.

According to the guideline of the National Strategy on Prevention and Control of Micronutrient Deficiencies (2015–2024), to strengthen effective coverage of IFA during pregnancy, every woman should get at least 90 IFA tablets during their first ANC and 90 at the subsequent check-up. Special packaging of IFA tablets to include a reminder tool and key messages for women should also be provided, and service providers should monitor for compliances in the subsequent ANC visits.^18^ Information from this study indicates that pregnant women were not getting IFA timely and in adequate quantity. Pregnant women were not getting IFA tablets before 5–6 months of their pregnancy, and they were getting 10–20 IFA tablets instead of 90 tablets. Service providers and programme managers also reported that they had to dispense fewer number of tablets as they had to cover many patients. Also, the medicine procurement and distribution process are very complex in Bangladesh. All community clinics in Bangladesh get the same number of medicines in a specific time of the year without considering the coverage areas of the community clinics. The purchase and distribution system involves different government health system departments (figures 1 and 2) and lack of coordination among the departments resulted in delays in procurement and supply of IFA tablets. A previous report on availability of medicine in government healthcare services indicated that availability of essential drugs was 39% at upazila health complex, and 17% at community healthcare centres. Also, only 28% of patients received all their drugs at the government facility, 58% patients received some of their medicines and 14% received no drugs at the facility.^38^ This study also reported that upazila and district hospitals had scarcity of medicine supply and most of the time some medicines were out of stock.^38^ In our study most of the Upazila Health and Family Planning Officer (UHFPO) and Upazila Family Planning Officer (UFPO) mentioned that they did not get the required amount of medicine in a timely manner which affected their service delivery. They emphasise on effective coordination among the primary service delivery centre and the CMSD which is aligned to the findings of a previous study that recommended establishment of proper requisition system from the hospital and a harmonised, functional, electronic drug management information system to monitor consumption, stock-out and expiry date.^38^

According to WHO, a sound and reliable information management system is the foundation of decision-making across all health system building blocks.^39^ In our study we found that maternal nutrition data were not routinely or systematically collected at the field level. The extent of reporting from the health and family planning departments were also limited. Also, the reporting format for collecting maternal nutrition-related data from Directorate General of Health and Directorate General of Family Planning were not updated accordingly.

Adequate nutrition during pregnancy is essential to reduce the risk of adverse birth outcome and improve the child’s nutritional status.^40^ However, our study findings indicate that more attention has been given to child nutrition than to the issues related to maternal nutrition. Similar scenario can be seen many in low- and middle-income countries where most of the programmes were focused on improving child nutrition.^13 18^

### Strengths and limitations

We included participants from both demand and supply sides to identify the barriers of nutrition service delivery in Bangladesh. This is the strength of our study. In addition, we included enough participants to reach a saturation point.

This study has few limitations as well. Although the KIIs and FGDs were carefully conducted, we do not know to what extent the participants over-reported or under-reported regarding the service delivery. We did not include the pregnant women who did not receive services from government facilities during data collection. Hence, we neither could reveal their perspective, nor we could assess the quality of services they received.

## Conclusion

This study was conducted to explore the constraints of implementing maternal nutrition-related interventions in Bangladesh. Beside these, we also gathered recommendations from our participants regarding successful delivery of maternal nutrition programmes in Bangladesh. The recommendation for the demand side includes increasing the awareness of husbands and mothers-in-law about the need to give nutritious diet to the women during pregnancy and lactation. For supply side, proper training on counselling pregnant women should be provided to primary healthcare providers; adequate doses of IFA and calcium supplementation must be ensured; national level indicators should be prepared for proper supervision and monitoring of maternal nutrition-related activities; country-specific intervention should be planned based on local context and global documents. Therefore, problem-specific interventions at the community, health facility and at the administrative levels should be implemented to address the identified barriers.

## Data Availability

Data are available upon reasonable request.
