# Supplementary material for: Qualitative assessment of programmatic constraints in delivery of effective interventions for improving maternal nutrition in Bangladesh
Source: BMJ Nutr Prev Health. 2023 Feb 15;6(1):65–75. doi: 10.1136/bmjnph-2021-000395 (PMC10407389; doi:10.1136/bmjnph-2021-000395)
Supplement: Supplementary data [file bmjnph-2021-000395supp001.pdf]

Supplementary table 1. Maternal nutrition related services provided by government service providers

Name of the organization	Name of the program	Maternal Nutrition Related activities under the program	Timeframe	Partners	Donors	Working Areas
Institute of Public Health and Nutrition (IPHN)	National Nutrition Services (NNS)	IFA supplementation<br>Calcium Supplementation<br>ANC-PNC services<br>Social and Behavioral Change Communication (SBCC)<br>➢ Message on nutritious food intake<br>➢ Promotion of ANC checkup	Ongoing (According to OP2017-2022)	Implemented by Upazila Health complex (UHC), at the root level through community clinic	Ministry of health and family welfare (MoHFW) with others development partner	National
DGFP	Maternal, Child, Reproductive and Adolescent Health (MCRAH)	ANC-PNC services<br>Deworming<br>IFA Supplementation<br>Nutrition message for pregnant women during ANC<br>• General food related message<br>• Importance of IFA and calcium	Ongoing (According PIP of 4th Health, Population and Nutrition Sector Program (HPNSP)	Implemented by Upazila family planning office at the root level through satellite clinic and Union Health and Family Welfare Centre (UH&FWC)	MoHFW with others development partner	National
Local Government Division	Urban Primary Health Care Services Delivery Project (UPHCSDP)	Pregnancy registration and follow-up<br>Clinical and pathological tests<br>ANC-PNC services<br>Nutritional message and counseling for women, husbands and other family members<br>• General food related message<br>• Importance of IFA and calcium	3rd phase(July 2012 to March 2018) 4th phase running (April 2018 to 2023)	Partner NGOs	ADB<br>GoB	11 city corporation and 14 municipalities areas.

Supplementary table 2: Maternal nutrition related services provided by non-government service providers

Name of the organization	Name of the program	Maternal Nutrition Related activities under the program	Timeframe	Partners	Donors	Working Areas
BRAC	Breast Feeding Support and Promotion	Breastfeeding support at Upazila Health Complex, Community and Workplace<br>Nutritional message and counseling for lactating mother<br>• General food related message<br>• Message on IFA and Calcium<br>**5 hospitals at Kurigram and 25 garment factories at Dhaka and Gazipur*	2016-2018	-	UNICEF	Kurigram<br>Dhaka<br>Gazipur
	Improving Maternal, Neonatal and Child Survival (IMNCS) (MNCH Rural)	Pregnancy identification and registration<br>ANC-PNC services<br>Promotion of IFA and deworming during pregnancy through home visit by the field worker (Shaysta Kormi)<br>Nutritional Message	2007-Ongoing	-	DFID, Ausaid	14 districts
	Manoshi (MNCH Urban)	ANC-PNC services<br>Nutritional Message<br>**Intervention is provided through 40 BRAC maternity centre and each center cover 1 lac to 1.20 lac population*	2007-Ongoing	-	DFID<br>Ausaid	Urban slums of 11 city corporation area
	(Maternal Infant and Young Child Nutrition) MIYCN	Provide food and nutrition related message to the pregnant mother to prevent anemia and child malnutrition.<br>provide Micronutrient supplementary powder for the pregnant women in Narshingdi and Manikganj	2017-2018	-	GAIN	Narshingdi and Manikganj
	Nutrition intervention pilot phase-2	Adolescent: School forum, Courtyard Counseling Session.<br>Pregnant Women: Counseling and Food Demonstration. Message on diversified food consumption and adequate food intake.<br>Social Mobilization: Fathers forum, Community Clinic	2016-2020	BRAC	SPA and DFID	141 upazilas

		Management and Support Group Meeting.				
	Targeting and Realigning Agriculture to Improve Nutrition (TRAIN)	Food demonstration through a food plate for the pregnant women.<br>IYCF related message,<br>Provide nutrition BCC and nutrition sensitive agricultural extension messages (targeted to both women and men.<br>Promote homestead gardening through the field worker. Provide technical support to the family for a homestead garden.	2016-2020	BRAC and IFPRI	Bill and Melinda gates foundation	Faridpur, Sherpur, Jamalpur, Kishoreganj, Narshingdi, Jhenaidah, Khulna, Gaibandha, Lalmonirhat, Rangpur.
	Jawtno	Conditional cash transfer for the pregnant women through electric money transfer system. Mother will get quarterly electronic cash transfers for utilizing services related to their children's health and development, including antenatal care visits, child growth monitoring and early learning activities for children under the age of five at the community clinics.<br>Maternal health and nutrition related message for the pregnant women and sensitize them to take service from the facility.	Ongoing	BRAC	World Bank	43 upazilas of Bangladesh.
Unicef collaboration with GOB	Nutrition Program	Training for capacity buildup for government facilities for providing maternal nutrition related service.<br>Help to develop accountability, reporting, training, monitoring and supervision for the government staff to provide maternal nutrition related service.<br>Provide medicine supply to government facilities.	Ongoing	DG health	Unicef	National
	MaMoni Maternal and Newborn Care Strengthening Project (MNCSP)	Strengthening the systems and standards for Maternal, Newborn, Child Health, Family Planning, and Nutrition (MNCH/FP/N) through policy regulatory issues<br>Improve the availability, quality and utilization of services in the public sector<br>Ensure nutrition services from facilities for the pregnant women and encourage mother to take service from the facilities by ensuring human resource support, performance	2013-2018	Local Partner NGOs	USAID	Habiganj,<br>Noakhali,<br>Lakshmipur,<br>Faridpur,<br>Manikganj,<br>Feni,

		monitoring and management.<br>Create awareness among the community people and provide nutrition message to the pregnant women through training of over 28000 skilled birth attendants and reaching over 80000 service providers, supervisors and managers in the public and private sectors.				Brahmanbaria<br>Chadpur.<br>Madaripur,<br>Kustia
Save the children	SUCHANA	Improve Nutrition Governance through enhance coordination within and between concerned ministries and sectors to increase resource allocation and effective imp<br>Improved access and utilization of nutrition services through build up capacity of local government institution to scale up nutrition specific and sensitive activities. .<br>Better nutrition through improved economic status through income generating activities and linked them with various government social protection schemes.<br>Social and behavior change communication<br>Generating robust evidence to support scale-up	2015-2022	Local partner NGOs and collaborators	European Union United Kingdom Department for International Development	Sylhet and Maulovibazar District
	C-MAMI	Improve facilities base nutrition services.<br>Training for the government service provider to ensure nutrition counseling for the pregnant women, child and lactating mother	2014- till date	Save the children	Margaret A. Cargill Foundation (MAC Foundation)	Barisal
	Improving Community Health Workers Program Performances through Harmonization & Community Engagement to Sustain Effective Coverage at Scale) ICHW	Establish efficient and effective linkages between communities, health and local systems to establish inclusive change in behavior that reduces gender barriers in system and social norms.<br>Capacity buildup for respective government facilities to provide nutrition support for the pregnant, lactating mother and newborn child.	2016-2020	MOHFW, MOLGRD, UNICEF, OGSB, BPA, BPMPA	The U.S. Agency for International Development (USAID)	Barisal

	Momota	Deployed health service providers (Medical Officer-2; Nurce-07; Paramedic-24) to government health facilities and ensure satellite clinic regularly, ensure 24/7 service from government health center.<br>Trained community volunteers for every 300 populations to Ensure health and nutrition related message and counseling for the pregnant women during ANC session.	2015-2017	FIVDB, DGHS and DGFP	KOICA and save the children	Sylhet
Care Bangladesh	Community Based Interventions To Improve Effective Coverage Of Maternal, New-Born And Child Health Services	Awareness creates among pregnant women to receive services from government facilities through ensuring home visit and arranged session by the government service provider (Health assistant).<br>Joint monitoring with the government to ensure quality services.	2016-2018	CARE Bangladesh.	UNICEF	Khulna
	Strengthening the Community Support System (CMSS) to Improve Maternal and Infant Health	Facilitate timely referral of women with obstetric complications to an appropriate EmOC facility.<br>Create awareness among the community about the danger signs of obstetric complications, available services at different facilities and values of proper rest and nutrition.	2015-2017	CARE Bangladesh		Kurigram
	Community Health Worker Initiative Phase II	Message on nutrition and diversified food during home visit.<br>Ensure 4 ANC for the pregnant women.<br>Follow up pregnant women to ensure institutional delivery	2015-2018	CARE Bangladesh	Glaxosmithkline (GSK)	Sunamganj
	Nutrition At The Center (N@C)	Training for the community health worker (Government) and community support group.<br>Community facilitator (CF) visits households, provide nutritional message (Importance of diversified food ad message about to consume IFA and calcium) and creates awareness on food diversity for the pregnant women.<br>Increase homestead production by providing technical support by the technical officer.	2013-2021	CARE Bangladesh	Private donor from the USA	Sunamganj
World vision Bangladesh	World Vision Bangladesh Area program	System strengthening of Health & Nutrition services (advocacy). Promotion of ANC/PNC and Institutional delivery. Promotion of additional food and care for PLW, home visit by	Ongoing	World Vision Bangladesh	WV fund	Sunamganj, Sylhet, Gazipur, Netrokona,

		community promoters and community facilitators				Chittagong, Rangpur, Dinajpur, Naogaon, Tangail Mymensingh, Bagerhat, Dhaka
	BRD-M&CN Project	Including increase the availability and diversity of food through home gardening.<br>Sensitize local people through community mobilization to overcome the gender barrier to increase access of nutritious food for women and children.<br>Medical Equipment's for local level health service delivery points like community clinic, family welfare center and Upazila health complex	Ongoing	World Vision Bangladesh	Korea International Cooperation Agency-KOICA	Jaypurhat, Naogaon
	Direct Nutrition Intervention Project	Health education session with the pregnant women on importance on IFA and calcium. Follow up to the pregnant women through the community volunteer (Service promoter)<br>Training for government service provider aims to capacity development to ensure quality maternal nutrition services at the community clinic<br>Tag the pregnant women with other development program to ensure nutrition-sensitive actions.	Ongoing	World Vision Bangladesh	UNICEF	Bhaluka (Mymensingh), Dacope and Koyra (Khulna)
	Nobojatra	Improved gender equitable food security, nutrition and resilience of vulnerable people<br>Conditional cash transfer for the pregnant women from second trimester to till the children become 9 months of age. ($27.5 per month)<br>Use mobile phone technology to send BCC message to the pregnant women and new mother.<br>Provide training to the government frontline service provider: Community health care provider (CHCP), Health assistant (HA), Family Welfare Assistant (FWA) so that they can provide quality MCHN service.	Sep 2015 to Sep 2020	World Vision Bangladesh	USAID, WFP and Winrock International (WI)	4 upazilas under two districts; Dacope, Koyra (Khulna); Shyamnagar, Kaliganj (Shatkhira)

	Enrich	Both Specific and Sensitive Nutrition Intervention including increase the availability and diversity of food through home gardening.<br>Sensitize local people through community mobilization to overcome the gender barrier to increase access of nutritious food for women and children.<br>Medical Equipment's for local level health service delivery points like community clinic, family welfare center and Upazila health complex	March 2016-September 2020	World Vision Bangladesh	Global Affairs Canada	Thakurgaon
(World food Program)<br>WFP	Improving Maternal and Child Nutrition	Supports the Government to reduce maternal and child under nutrition, Improving the nutritional status of mother and child, ensures community members become actively involved in improving nutrition outcomes, referral system for the severe undernourished child, receive oil and Super Cereal, a Wheat soya flour mixed with sugar and micronutrients, BCC on IYCF, hygiene and sanitation practices.	Ongoing	Bangladesh Government.	WFP	Kurigram, Gaibandha, Sirajganj, Dhaka, Satkhira,<br>Cox Bazar
FHI 360	Alive and thrive<br>Demonstrating the feasibility of integrating maternal nutrition in MNCH	To increase dietary diversity and intake of energy, iron, folic acid, and calcium for the pregnant women. (Home visit through Systho Kormi_SK)<br>SBCC activities through community mobilization with Husband's Forum, Union Parishad orientation, and orientation of formal and informal health care providers<br>Orientation of formal and informal health care providers, village doctors/local pharmacists, religious leaders, local leaders/elites, and teachers to create awareness on maternal nutrition activities.<br>Create broad public awareness about maternal nutrition in media dark areas, village/street theater troupes were hired to raise public awareness about desirable nutrition practices.	2008-2022	BRAC	Bill & Melinda Gates Foundation	BRAC MNCH program Kurigram, Lalmonirhat, Mymensingh, and Rangpur
Nutrition International	Right Start	Provide training to build the capacity of healthcare service providers to promote and deliver interventions to improve care for pregnant mothers, reducing risks to their health and	2015-2020	Strategic Partner DGHS, DGFP and NNS	Global Affairs Canada (GAC)	Dinajpur, Rajshahi, Pabna, Tangail, Gazipur,

		the health of their newborns.<br>Provide IFA tablets,<br>Increase accessibility of fortified rice for the women of reproductive age and adolescent girls through government safety net program.				Hobiganj, Munshiganj,Comilla, Bhola, Bagerhat district, Khulna and Gazipur city coorporation
	Maternal nutrition (TB-14 Grant of Canadian government)	Provide IFA tablets, Supply delivery and stock maintain of IFA tablet. Help civil surgeon, UNHPO and UFPO in supply chain management which helps to maintain smooth supply of IFA in the UHC and Community clinic.	2014-2019	NI with the collaboration with NNS	Canadian Government	Gaibandha, Jamalpur, Barisal, Jessore, Sunamganj, Kishoreganj, Noakhali, Bogra, Sirajganj, Sherpur, Kustia, Chuadanga, Meherpur, Madaripur, Feni, Joypurhat, Natore, Barguna, Jhalokati, Narail
CARITAS Bangladesh	USAID's Improving Nutrition Through Community Approaches (INCA)	Nutrition counseling, food demonstration for the pregnant, lactating mother, child and adolescent girls.<br>Provide training to develop capacity for provide better nutrition services from government facilities.<br>Promote ANC and PNC. SBCC activities with the pregnant women with the help of community service provider. .		CARITAS and United purpose.	USAID	Noakhali, Lakshmipur, Bhola
Helen Keller International (HKI)	SAPLING	Increased income and access to nutritious foods attained equitably by both women and men by increasing homestead food production.<br>Improved nutritional status of children under five years of age, pregnant and lactating women and adolescent girls; and Sustained gender equitable ability of people, households,	2015-2020	Catholic Relief Services (CRS), and Caritas/Bangladesh	USAID	Bandarban

		communities, and systems to mitigate, adapt to and recover from human-induced and natural shocks and stresses through nutrition education session. This session is implementing through courtyard session and one to one counseling.				
	FIRM	Provide maternal nutrition education for the pregnant women. (Nutrition sensitive and specific message, Diversified food consumption, IFA and calcium consumption, create awareness about 4 ANC, gender issues on food consumption.)<br>They are working to increase homestead production and food diversity within the program area. Increase homestead production by establish vegetable garden, cattle raring	2014-2017	HKI and voluntary association for rural development (VIRD)	USAID	Mowlovibazar
Action Contre la faim	Multi - sartorial approach to reduce malnutrition in Bangladesh	ACF handed over community-based management of acute malnutrition (CMAM) to local actors with whom ACF has worked for several years. ACF continues to provide technical support to health authorities in the district, by monitoring and supervising activities.	2016- 2017	Shushilan	ACF<br>ECHO<br>UNICEF<br>UNHCR<br>BPRM (USA)<br>WFP	Shatkhira
Terre des Homes (TDH)	Mother and child health program	Fights against maternal and child malnutrition by organizing awareness campaigns, seminars, discussion groups and cooking demonstrations. TDH helps to increase food production is the creation of elevated gardens, which are less prone to flooding. TDH works with the local authorities and medical staff to improve existing facilities and the quality of care for children.	Ongoing	Terre des homes	Swiss Solidarity, DDC - DEZA – SDC, Terre des homes Netherlands, FEDEVACO, Canton of Geneva, UNICEF	Kurigram
Bangladesh Extension Education Services (BEES)	SHEBA- Health Program	Anti-natal care and post-natal care. Counseling the mothers about colostrum feeding, exclusive breast feeding.<br>Nutrition education for the PLW, adolescent girls.<br>provide vegetable seeds, seedlings and saplings to establish	2002- till date	Bangladesh Extension Education Services	Self-Funded	Narsingdi, Kishorganj, Bogra, Gaibandha & Rangpur districts.

		small homestead gardens for maintaining nutritional status of target beneficiaries.		(BEES)		
NGO Health Services Delivery Project (NHSDP)	Smiling Sun	providing technical assistance to further develop and expand current nutrition services, improve quality of service provision, and increase coverage of services through<br>• Supporting nutrition training for service providers for improved delivery of nutrition services<br>• Strengthening quality improvement and quality assurance through the development of tools and through supportive supervision<br>• Improving monitoring and evaluation to strengthen nutrition service delivery<br>• Assessing improvements in NHSDP's nutrition service delivery following enhanced activities	2012- till date (Current Phase)	Chamonics International with more than 25 local NGOs	USAID, DFID, Chevron	National
